# Cardiovascular Risk Prediction in Older Adults

**DOI:** 10.1007/s11883-025-01339-2

**Published:** 2025-09-09

**Authors:** Lisandro D. Colantonio, Vera Bittner

**Affiliations:** 1 Department of Epidemiology, University of Alabama at Birmingham, Birmingham, AL USA; 2 Department of Medicine, Division of Cardiovascular Disease, University of Alabama at Birmingham, 521 19th Street South-GSB 444, Birmingham, AL 35233 USA

**Keywords:** *[MeSH terms]*: cardiovascular disease, Risk assessment, Risk factors, Heart disease risk factors, Aged

## Abstract

**Purpose of Review:**

This review examines cardiovascular disease (CVD) risk prediction models relevant to older adults, a rapidly expanding population with elevated CVD risk. It discusses model characteristics, performance metrics, and clinical implications.

**Recent Findings:**

Some models have been developed specifically for older adults, while several others consider a broader age range, including some older individuals. These models vary in terms of predictors, outcomes, horizon, and statistical approaches, with some accounting for competing risks and considering age-predictor interactions. Discrimination is generally acceptable and more modest in older versus younger individuals. Calibration shows great variation across populations.

**Summary:**

Accurate CVD risk prediction is essential to guide individualized prevention strategies and support shared decision-making in older adults. CVD risk prediction in this population is challenged by age-related CVD risk heterogeneity, elevated competing risk due to non-CVD mortality, and comorbidities. Further refinement by incorporating geriatric-specific factors may help to enhance discrimination.

## Introduction

The world population is aging rapidly [1]. Between 2015 and 2050, the percentage of people aged 65 and older is expected to nearly double, from 8.5 to 16.7% [1]. This demographic shift poses a significant challenge for healthcare systems, which must adapt to the increased demand for the prevention and management of chronic conditions that are more common in older adults, including cardiovascular disease (CVD) [2]. CVD prevention and management are commonly guided by the predicted risk of CVD [3–6]. However, most CVD risk prediction models recommended for use in current guidelines were developed excluding very old individuals [4–9], and these models typically perform worse in older compared to younger adults [10–15]. This represents a critical limitation for the prevention and management of CVD in older adults, which needs to be addressed.

Several challenges exist in predicting CVD risk in older adults. Prior studies have shown that the relative risks for CVD associated with some traditional risk factors like diabetes, hypertension, obesity, and high cholesterol, are lower in older versus younger adults, while the absolute CVD risk in those with these risk factors is higher with older age [16–20]. Also, older adults have high mortality and, without accounting for non-CVD-related deaths that prevent future CVD events from occurring (i.e., a situation referred to as “competing risk” in the literature), predicted CVD risk may be overestimated [16, 21, 22]. Finally, older adults frequently experience geriatric conditions like sarcopenia, frailty, depression, or cognitive impairment, which may increase the risk of CVD or its competing risks [16, 23, 24]. These geriatric conditions are often overlooked in CVD risk prediction models recommended for use in current guidelines [4–8].

The purpose of this review is to describe CVD risk prediction models for older adults, typically defined as those ≥ 65 years of age [25]. Specifically, we describe the publication year, country or region, target population, outcome components, horizon, predictors, statistical model characteristics, and discrimination. The calibration of CVD risk prediction models is very sensitive to the population analyzed [26]. Therefore, we describe results on calibration only when these are from an external validation. Table 1 shows some key terminology and definitions used in the current review, including discrimination, calibration, and validation [21, 22, 27–29]. We describe first CVD risk prediction models limited to older adults. For completeness, we also describe CVD risk prediction models that include middle-aged adults and some older age groups. Most CVD risk prediction models are developed for primary prevention. CVD risk prediction models for secondary prevention are described separately.

**Table 1:** Key terminology and definitions used in the current review

Terminology	Definition
Calibration	It is a model performance measure that refers to the degree to which the absolute predicted risk value from a risk prediction model agrees with the observed or true risk [27]. Models can have good calibration, underpredict, or overpredict risk.
Competing risks	It refers to those events, most often death, which will prevent future occurrences of the outcome of interest [21, 22].
Discrimination	It is a model performance measure that refers to the ability to distinguish between individuals who will have the event versus those who will not [27]. Models can have good or poor discrimination, and this is different from calibration. For example, a model may overpredict risk and still give higher risk values to those more likely to have the event.
Horizon	It refers to the period over which the model predicts risk. Horizon is important to consider because the absolute risk value will be higher with a longer follow-up.
Outcome	It refers to the specific event of interest that the model predicts. The current review focuses on risk prediction models that include a variety of types and forms of CVD events (e.g., coronary heart disease, cerebrovascular disease, fatal or nonfatal).
Predictors	It refers to the list of characteristics or behaviors included in a risk prediction model and used to calculate the risk. These characteristics and behaviors are often referred to as “risk factors”.
Prevention, primary	Refers to the care of individuals without a history of CVD. Risk prediction models for primary prevention will predict the risk of a first or incident CVD event.
Prevention, secondary	Refers to the care of individuals with a history of CVD. Risk prediction models for secondary prevention will predict the risk of a recurrent or subsequent CVD event.
Risk prediction model	It is a tool, often in the form of mathematical equations or formulas, that can be used to estimate a person’s risk for experiencing an outcome of interest over a specific horizon based on some known predictors.
Validation, external	It is a type of analysis aimed at assessing the performance of a risk prediction model using a different dataset from which the model was developed [28].
Validation, internal	It is a type of analysis aimed at assessing the performance of a risk prediction model using the same dataset (or a subset of it) from which the model was developed [29].

*CVD* Cardiovascular disease

## Summary

### CVD Risk Prediction Models for Primary Prevention in Older Adults

Table 2 presents CVD risk prediction models for primary prevention specific for older adults [24, 30–38]. These 10 models were developed for countries in Europe, the United States (US), Australia, Taiwan, and China. A total of five models have been published since 2020, including ASPREE (Australia/US, 2022), CHARLS (China, 2025), NTUH-iMD (Taiwan, 2025), SCORE2-OP (Europe, 2021), and SENIOR (US, 2025).

**Table 2:** CVD risk prediction models for primary prevention limited to older adults

Model / Year	Country / Region	Target population	Outcome (components)	Horizon	Predictors	Statistical model	Discrimination	Calibration (external)
ANBP2 / 2015 [30]	Australia	Adults 65 to 84 years of age without a history of CVD with hypertension	CVD death from the Australian National Death Index	10-year	Age, sex, smoking, alcohol consumption, physical activity, diabetes, waist-hip ratio, disadvantaged socioeconomic status	Cox regression	C-statistic: 0.707 (internal validation); 0.729 (external validation in the Dubbo study [Australia])	Calibration was considered acceptable based on plots, but it is unclear whether these were from the external validation
ASPREE / 2022 [24]	Australia, US	Adults ≥70 years of age without a history of CVD, dementia, or physical disability	MACE (MI, CHD death, or fatal or nonfatal stroke)	5-year	Age, sex, smoking, SBP, HDL and non-HDL cholesterol, creatinine, diabetes, and antihypertensive medication use	Cox regression	AUC (external validation in the Predict cohort, New Zealand): 0.64 (95% CI 0.63, 0.66)	Underpredicted risk in PREDICT (New Zealand)
CHARLS / 2025 [31]	China	Adults ≥60 years of age without heart disease with hypertension	Reported heart disease	7-year	Age, gender, waist-to-height ratio, smoking, alcohol consumption, physical activity, dyslipidemia, diabetes, malignant tumors, chronic lung diseases, heart and liver diseases, stroke, kidney diseases, digestive system disorders, emotional and mental health issues, memory-related diseases, arthritis or rheumatic diseases, and asthma	XGBoost, DNN, and logistic regression	AUC (internal validation): logistic regression 0.60 (95% CI: 0.53, 0.68), XGBoost 0.64 (95% CI: 0.57, 0.71), DNN 0.67 (95% CI: 0.60, 0.73)	External validation unavailable
CHS / 2013 [32]	US	Adults ≥65 years of age without clinical CVD with diabetes	CVD (MI, stroke, and CVD death)	10-year	Age, smoking, SBP, total and HDL cholesterol, creatinine, use of glucose-lowering agents, C-reactive protein, ankle–brachial index, electrocardiographic left ventricular hypertrophy and internal carotid intima–media thickness	Cox regression. Sex-stratified model. Considered interactions between age and risk factors.	C-statistic (external validation in MESA): 0.68	The model was recalibrated for the external validation in MESA
CHS-RS / 2012 [33]	The Netherlands / US	Adults ≥65 years of age without a history of CVD	CHD (nonfatal MI fatal MI, atherosclerotic CHD death)	10-year	Age, smoking status, diabetes, SBP, antihypertensive medication, and total and HDL cholesterol	Fine and Gray model, accounts for the competing risk of non-CHD death. Sex-specific models.	C-statistic (internal validation): 0.63 in both US and European men; 0.68 and 0.67 in US and European women, respectively	External validation unavailable
NTUH-iMD / 2025 [34] (single hospital cohort)	Taiwan	Adults ≥75 years of age without a history of CVD	CVD (MI, stroke or CVD death)	5-year risk	For men: for men comprised advanced age, smoking, non-HDL cholesterol >160 mg/dL, metastatic cancer, and aspirin usage. For women: advanced age, smoking, atrial fibrillation, cancer, dementia, osteoarthritis, systemic lupus erythematosus, use of antihypertensives, and use of oral anticoagulants	Cox regression. Sex-specific models. Considered interactions between age and risk factors.	AUC (internal validation): 0.64 in men; 0.68 in women	External validation unavailable
preDIVA / 2019 [35]	The Netherlands	Adults 70-78 years of age without a history of CVD or dementia	CVD (CVD death, MI, stroke, TIA, angina, or PAD)	5-year	Age, sex, smoking, SBP, total and HDL cholesterol, diabetes, polypharmacy, symptoms of apathy	Four different models including different predictors, accounting (Fine and Gray) and not accounting (Cox regression) for the competing risk of non-CVD death.	C-index range (internal validation): 0.63-0.65 across models	External validation unavailable
PROSPER / 2017 [36]	Northen Europe	Adults 70-82 years of age without a history of CVD or chronic kidney disease stages IV or V	MACE (MI, stroke or CVD death)	5-year and 10-year	Sex, age, smoking, diabetes, SBP, LDL and HDL cholesterol, eGFR, number of medications taken, and statin treatment	Fine and Gray model, accounting for the competing risk of non-CVD death	C-statistic (external validation in the ASCOT-LLA trial): 0.57 (95% CI 0.53, 0.63)	The model was recalibrated for external validation in the ASCOT-LLA
SCORE2-OP / 2021 [37] (derived using CONOR)	Europe, divided into four CVD risk regions	Adults ≥70 years of age without a history of CVD	Primary: CVD (MI, stroke, or CVD death) Secondary: CVD (MI, stroke, HF hospitalization, or CVD death)	5-year and 10-year	Age, smoking, diabetes, SBP, and total and HDL cholesterol	Fine and Gray models accounting for the competing risk of non-CVD death. Sex-specific models. Considered interactions between age and risk factors	C-index (external validation across cohorts): Primary outcome: range 0.63-0.67 Secondary outcome: range 0.63-0.67	The model was recalibrated for external validation
SENIOR / 2025 [38] (derived using ARIC, CHS, and Framingham original cohort, exam 24)	US	Adults ≥75 years of age without a history of CVD	CVD (CVD death, MI or stroke)	5-year	SBP, HDL cholesterol, employment, triglycerides, creatinine, mobility, Mini-Mental State Examination, use of antihypertensive medication, lipid-lowering medication and aspirin	Fine and Gray models accounting for the competing risk of non-CVD death	C-index: Internal validation: 0.67 (95% CI 0.62, 0.71) External validation: 0.59 (95% CI 0.53–0.66)	Substantially overpredicted risk in the external validation in MESA

*ANBP2* Second Australian National Blood Pressure study, *ARIC* atherosclerosis risk in communities, *ASCOT*-*LLA* Anglo-Scandinavian Cardiac Outcomes Trial-Lipid Lowering Arm, *ASPREE* ASPirin in Reducing Events in the Elderly study, *AUC* area under the curve, *CHARLS* China Health and Retirement Longitudinal Study, *CHD* coronary heart disease, *CHS* Cardiovascular Health Study, *CI* confidence interval, *CONOR* cohort of Norway, *CVD* cardiovascular disease, *DNN* Deep Neuronal Network, *eGFR* estimated glomerular filtration rate, *HDL* high-density lipoprotein, *HF* heart failure, *LDL* low-density lipoprotein, *NTUH*-*iMD* National Taiwan University Hospital-integrative Medical Database, *MACE* major adverse cardiovascular events, *MESA* Multi-Ethnic Study of Atherosclerosis, *MI* myocardial infarction, *PAD* peripheral artery disease, *preDIVA* Prevention of Dementia by Intensive Vascular care, *PROSPER* Prospective Study of Pravastatin in the Elderly at Risk, *RS* Rotterdam Study, *SBP* systolic blood pressure, *SCORE*-*OP* Systematic COronary Risk Evaluation Older Persons, *US* United States, *XGBoost* Extreme Gradient Boosting

Most risk prediction models (*n* = 7) do not consider an upper age limit and, therefore, could be applied to the oldest old. However, whether these studies included an adequate representation of very old individuals is unclear. ANBP2 and CHARLS were restricted to adults with hypertension (a common comorbidity in older adults) [30, 31], while CHS was restricted to those with diabetes [32]. ASPREE, preDIVA, and PROSPER excluded older adults with dementia, physical disability, and chronic kidney disease, respectively [24, 35, 36].

The outcomes predicted include CVD (CHS, NTUH-iMD, preDIVA, SCORE2-OP, SENIOR), CVD death (ANBP2), major adverse cardiovascular events (ASPREE, PROSPER), and coronary heart disease (CHS-RS). CHARLS (China) predicts the risk for incident heart disease defined by the report of participants or family members [31]. This model used data from adults ≥ 60 years of age with hypertension collected in 2011 and 2018 (7 years apart). All other models considered a horizon of 5 or 10 years for risk prediction, with some models allowing the calculation of both.

Commonly used predictors include traditional risk factors like age, sex, smoking, diabetes, blood pressure, and cholesterol. Antihypertensive medication, lipid-lowering medication, and aspirin use are incorporated in some risk prediction models. Less frequently used predictors include: disadvantaged socioeconomic status, employment, alcohol consumption, physical activity, mobility, waist-hip ratio, waist-to-height ratio, C-reactive protein, triglycerides, number of medications taken, polypharmacy, atrial fibrillation, estimated glomerular filtration rate (eGFR), creatinine, kidney disease, dementia, memory-related diseases, cancer, malignant tumors, osteoarthritis, oral anticoagulation, rheumatic diseases, asthma, chronic lung disease, liver disease, digestive system disorders, ankle-brachial index, electrocardiographic left ventricular hypertrophy, internal carotid intima-media thickness, emotional and mental health, and Mini-Mental State Examination.

CHS-RS, preDIVA, PROSPER, SCORE2-OP, and SENIOR were derived using time-to-event regression accounting for the competing risk of non-outcome-related deaths [33, 35–37]. ANBP2, ASPREE, CHS, and NTUH-iMD were derived using Cox proportional hazards regression, which does not account for competing risks [24, 30, 32, 34]. CHARLS (China) used machine learning methods and logistic regression to estimate the absolute risk at 7 years [31]. The discrimination of the models was generally modest, and only ANBP2 had a C-statistic > 0.70 (C-statistic of 0.729 from the external validation in the Dubbo study, another Australian cohort) [30].

Information on the calibration of risk prediction models based on external validations is limited. Authors of the ANBP2 (Australia) reported an acceptable calibration based on plots, although it is unclear whether the calibration was assessed internally or externally [30]. ASPREE (Australia/US) underestimated the observed risk among adults ≥ 70 years of age in PREDICT (New Zealand), and the authors considered that recalibration may be necessary [24]. SENIOR (US) substantially overpredicted risk in the Multi-Ethnic Study of Atherosclerosis (MESA), which may be due to the use of old cohorts’ data for its derivation [38]. No external validation was available for the NTUH-iMD model, but this model overpredicted the observed CVD risk at 5 years in the internal validation, particularly among men with low/moderate predicted risk [34]. Other models were recalibrated before external validation, including CHS [32], PROSPER [36], and SCORE2-OP, which was calibrated to European countries classified into four CVD risk regions [37]. A validation study using the SCORE2-OP for low-risk countries found an underprediction of risk among women (expected-to-observed ratio 0.77) and a slight overestimation among men (expected-to-observed ratio 1.06) in a sample of primary care patients from Barcelona [39]. Although the SCORE2-OP has been externally validated using data from the US, North Africa, China, and Australasia [37], the impact of using this risk prediction model for clinical decision-making in these regions remains unclear. CHS (US), CHS-RS (the Netherlands / US), ASPREE (Australia / US), and SENIOR (US) included mostly white adults [24, 32, 33]. Therefore, the applicability of these risk prediction models to non-white US populations is unclear.

### Other CVD Risk Prediction Models Including Older Adults

Table 3 presents CVD risk prediction models for primary prevention that included older adults in their derivation [40–67]. Some of these models do not report an upper age limit (JALS, RRS, and SCORE2-Diabetes [53, 61, 62, 64]), or this is very high (90 years for the Hisayama study and LIFE-CVD2 [50, 54]). These models could be applied to all or most older individuals. However, caution is needed as some of these models may have included a small number of very old individuals in their derivation. Therefore, the predicted CVD risk in very old adults may not be accurate, particularly if interactions between age and risk factors were not considered.

**Table 3:** CVD risk prediction models for primary prevention that include some older adults

Model / Year	Country / Region	Target population	Outcome (components)	Horizon	Predictors	Statistical model	Discrimination	Calibration (external)
ASSIGN 2.0 / 2025 [40]	Scotland	Adults 40–69 years of age without a history of CVD	CVD (CHD, stroke, TIA, coronary or carotid revascularization, CVD death)	10-year	Age, sex, total and HDL cholesterol, SBP, diabetes, family history of CVD, number of cigarettes per day, and the Scottish Index of Multiple Deprivation score	Cox regression. Sex-stratified model. The model was derived from recalibrating the prior ASSIGN equations	C-index (external validation): 0.682 (95% CI 0.679, 0.686) in men; 0.706 (95% CI 0.702, 0.710) in women	Calibration slopes: Men: 0.999; Women: 0.900
Aus CVD Risk / 2023 [41–43]	Australia	Based on the PREDICT model (New Zealand), calibrated to adults 30–79 years of age without a history of CVD in Australia.						
China-PAR equations / 2016 [44]	China	Adults 35 to 74 years of age without a history of MI or stroke	CVD (MI, CHD death, fatal and nonfatal stroke)	10-year	Age, sex, smoking, waist circumference, geographic region, diabetes, SBP, antihypertensive medication, total and HDL cholesterol, rural-urban residence, family history of CVD	Cox regression. Sex-stratified model. Includes interaction terms between age and other risk factors	C statistic (external validation): Men range 0.793–0.809 Women range 0.805–0.829	Calibration was considered acceptable based on calibration χ^2^ (13.1 in men and 12.8 in women)
EPOCH-JAPAN / 2021 [45]	Japan	Adults 40–79 years of age without a history of CVD	Death from CHD, stroke, and CVD, separately (three different models)	10-year	Age, sex, smoking, SBP, proteinuria, diabetes, total-to-HDL cholesterol ratio. Interactions with age are included.	Cox regression. Includes interaction terms between age and other risk factors	C-indices (internal validation): CHD model: 0.83; Stroke model: 0.80; CVD model: 0.81	External validation unavailable
Framingham general CVD risk score / 2008 [46]	US	Adults 30–74 years of age without a history of CVD	Total CVD (CHD death, MI, coronary insufficiency, angina, ischemic and hemorrhagic stroke, TIA, intermittent claudication, and heart failure	10-year	Age, sex, total and HDL cholesterol, SBP, antihypertensive medication use, diabetes and smoking	Cox regression. Sex-stratified model.	C-statistic (internal validation): Men 0.763 (95% CI 0.746, 0.780) Women 0.793 (95% CI 0.772, 0.814)	20% CVD risk overprediction in older adults in the ASPREE extension trial [70]
Globorisk / 2015–2022 [47–49]	Worldwide 182 countries	Adults 40–74 years of age without a history of CHD or stroke	CVD (MI, CHD death, fatal or nonfatal stroke, sudden cardiac death)	10-year	Lab risk charts: Age, sex, smoking, SBP, diabetes and total cholesterol Office risk charts: Age, sex, smoking, SBP and BMI	Cox regression. Age and sex specific charts. Age is used as the time scale. Includes interactions between age and risk factors	Harrell’s C statistics (external validation): Lab risk charts: range 0.73–0.78 Office risk charts: range 0.70–0.77	Models were recalibrated using age-sex mean risk factor levels and CVD rates in each country. The lab risk Globorisk-LAC model overestimated risk in Colombia [99]
Hisayama Study / 2009 [50]	Japan	Adults ≥ 40 years of age without a history of CVD	CVD (CHD or stroke)	10-year	Age, sex, SBP, diabetes, LDL and HDL cholesterol, smoking	Cox regression.	C-statistic (internal validation): 0.81 (95% CI: 0.77, 0.86)	External validation unavailable
IHRS / 2011 [51, 52]	Worldwide	Adults 35–70 years of age	CVD (MI, stroke, HF, revascularization, and CVD death)	Non-determined	Non-laboratory model: age, sex, parental history of CHD, diabetes, hypertension, smoking, second-hand smoking, low-physical activity, depression, waist-hip ratio, diet Laboratory model: age, sex, apolipoprotein B: A1 ratio, smoking, diabetes, hypertension	Logistic regression (developed using a case-control design)	C-statistic (internal validation in 2011): 0.67–0.79 across regions	Models were recalibrated in 2018 using prospective data [52]. The non-laboratory model underpredicted risk in Colombia [99]
JALS / 2019 [53]	Japan	Adults 40–90 years of age without a history of stroke or heart disease	Stroke, MI, composite of stroke or MI, and CVD death, separately	5-year 10-year	Age, sex, diabetes, smoking, blood pressure, antihypertensive medication, HDL and non-HDL cholesterol, BMI, eGFR, AF (predictors vary by outcome)	Poisson regression model (incidence rate).	AUC (internal validation): Stroke: 0.772, MI: 0.814, stroke or MI: 0.771, CVD death: 0.832	External validation unavailable
LIFE-CVD2 / 2024 [54] (derived using individual-participant data from 44 cohorts in 13 countries)	Europe (four risk regions)	Adults 35–90 years of age without a history of CVD (models are not intended for individuals with diabetes)	CVD (MI, stroke, or CVD death)	Lifetime	Age, sex, smoking, diabetes, SBP, and total and HDL cholesterol	Accounts for the competing risk of non-CVD death by modeling the CVD risk and non-CVD risk, separately, using Cox regression. Sex specific models. Uses age as the timescale. Includes interactions between age and risk factors	C-index (external validation in eight cohorts): 0.795 (95% CI 0.767–0.822)	The model was recalibrated to four risk regions in Europe. Overall, good calibration in two external cohorts from UK and The Netherlands.
NORRISK2 / 2017 [55] (derived using data from CONOR)	Norway	Adults 40–79 years of age without a history of CVD	MI or stroke	10-year	Age, sex, total and HDL cholesterol, SBP, antihypertensive medication use, smoking, family history of CHD	Accounts for the competing risk of non-CVD death. Age and sex specific charts.	AUC (external validation): 0.62 (95% CI 0.59, 0.64) in men 65–74 years 0.64 (95% CI 0.60, 0.68) in women 65–74 years. For comparison, the AUC for men and women was 0.65 (95% CI 0.62, 0.68) and 0.68 (95% CI 0.65, 0.72), respectively in adults 55–64 years of age, and 0.70 (95% CI 0.65, 0.74) and 0.68 (95% CI 0.62, 0.75) in those 45–54 years of age	Good agreement based on calibration plots
PCE / 2014 [56]	US	Adults 40–79 years of age without a history of CVD	Atherosclerotic CVD (nonfatal MI, CHD death, fatal or nonfatal stroke)	10-year	Age, sex, race, total and HDL cholesterol, SBP, antihypertensive medication use, diabetes, smoking	Cox regression. Sex and race specific models.	C-statistics (internal validation): White women: 0.818 White men: 0.757 African-American women: 0.759 African-American men: 0.713 Discrimination was lower (C-statistic 0.62) in older adults [14]	Overestimated risk among older adults [14]
PREDICT / 2018 [57]	New Zealand	Adults 30–74 years of age without a history of CVD or renal disease	Total CVD (CHD (including angina), ischemic and hemorrhagic cerebrovascular events (including TIA), peripheral vascular disease, HF, and other ischemic CVD death	5-year	Age, sex, ethnicity, family history of premature CVD, socioeconomic deprivation, smoking, diabetes, SBP, total-to-HDL cholesterol ratio, AF, antihypertensive medication, lipid-lowering medication, and antithrombotic medication use.	Cox regression. Sex-specific models.	Harrell’s C statistic (internal validation): Men 0.73 (95% CI 0.72–0.73) Women 0.73 (95% CI 0.72–0.73)	External validation unavailable
PREVENT / 2023 [58, 59]	US	Adults 30–79 years of age without a history of CVD	Primary: CVD (atherosclerotic CVD or HF) Secondary: atherosclerotic CVD (CHD or stroke) and HF, separately	10-year and 30-year	Age, sex, smoking, BMI, SBP, antihypertensive medication use, HDL and non-HDL cholesterol, statin use, diabetes, and eGFR Optional: uACR, HbA1c, and social deprivation	Accounts for the competing risk of non-CVD death. Sex specific, race free. Age was modeled as the time scale. Includes interactions between age and risk factors	Median C-statistics for CVD, ASCVD, and HF (external validation): 0.794, 0.774, and 0.830 in women, respectively 0.757, 0.736, and 0.809 in men, respectively	Calibration slopes for CVD, ASCVD, and HF: 1.03, 1.09, and 1.00 in women, respectively 0.94, 1.04, and 0.89 in men, respectively. Good calibration in Kaiser Permanente Southern California [100]
QRISK3 / 2017 [60] (derived using data from primary care patients in England)	England	Adults 25–84 years of age	CVD (CHD, ischemic stroke or TIA)	10-year	Age, sex, race, smoking, diabetes, SBP, antihypertensive medication, total-to-HDL cholesterol ratio, SBP variability, BMI, corticosteroid use, CKD, AF, erectile dysfunction (men), migraine, rheumatoid arthritis, SLE, severe mental illness, atypical antipsychotic use, material deprivation, family history of CHD in first degree relative < 60 years of age	Cox regression. Sex stratified models. Includes interactions between age and risk factors	Internal validation: Harrell’s C statistic in men and women ≥ 60 years of age 0.692 (95% CI 0.689, 0.695) and 0.659 (95% CI 0.656, 0.663), respectively. For comparison, the Harrell’s C statistics were 0.752 (95% CI 0.747, 0.757) and 0.732 (95% CI 0.728, 0.736), respectively, for participants 40–59 years of age, and 0.747 (95% CI 0.728, 0.766) and 0.781 (95% CI 0.771, 0.792) in those < 40 years of age. External validation in the UK Clinical Practice Research Datalink [68]: Harrell’s C statistic < 0.65 in all subgroups ≥ 65 years of age	Overpredicted risk in adults 75–84 years of age in the UK Clinical Practice Research Datalink [68]
RRS women / 2007 [61]	US	Women ≥ 45 years of age without a history of CVD	CVD (MI, stroke, coronary revascularization, or CVD death)	10-year	Age, total and HDL cholesterol, SBP, smoking, CRP, diabetes and hemoglobin A1C, parental history of MI before age 60 years	Cox regression. Sex specific model.	C-statistic 0.8 (internal validation)	External validation unavailable
RRS men / 2008 [62]	US	Men ≥ 50 years of age without a history of CVD or diabetes	CVD (MI, stroke, coronary revascularization, or CVD death)	10-year	Age, total and HDL cholesterol, SBP, smoking, CRP, parental history of MI before age 60 years	Cox regression. Sex specific model.	Harrol’s C-Index 0.71 (internal validation)	External validation unavailable
SCORE2 / 2021 [63] (derived using individual-participant data from 45 cohorts in 13 countries)	Europe (divided into four risk regions)	Adults 40–69 years of age without a history of CVD	CVD (MI, stroke, or CVD death)	10-year	Age, sex, smoking, diabetes, SBP, and total and HDL cholesterol	Accounts for the competing risk of non-CVD death. Sex specific models. Includes interactions between age and risk factors	C-index (external validation): 0.67–0.81 across different cohorts	The model was recalibrated to four risk regions in Europe
SCORE2-Diabetes / 2023 [64]	Same as SCORE2	Adults ≥ 40 years of age with type 2 diabetes and without a history of CVD	Same as SCORE2	10-year	Extended SCORE2 models by adding diabetes-related variables: HbA1c, age at diabetes diagnosis, and eGFR	Same approach as SCORE2	C-index (external validation): 0.658–0.688 across 4 cohorts (improved discrimination versus SCORE2)	The model was recalibrated to four risk regions in Europe using recalibration factors from SCORE2 and SCORE2-OP. An external validation in the Netherlands showed good calibration in the Dutch population, and underestimation in the non-Dutch population [65]
Suita Study / 2020 [66]	Japan	Adults 30–79 years of age without a history of CVD	CVD (CHD or stroke)	10-year	Age, sex, blood pressure, HDL, non-HDL, and LDL cholesterol, diabetes, smoking, and proteinuria. A second model also includes AF and LVH by ECG	Cox regression.	C-statistics (internal validation): With ECG: 0.782 (95% CI 0.766–0.799); Without ECG: 0.781 (95% CI 0.765, 0.797)	External validation unavailable
WHO / 2019 [67]	Worldwide 21 WHO regions	Adults 40–80 years of age without history of CVD	CVD (MI, CHD death, and stroke)	10-year	Laboratory-based model: age, smoking, SBP, diabetes, total cholesterol. Non-laboratory-base model: age, smoking, SBP, BMI	Cox regression. Sex-specific models. Includes interactions between age and other risk factors.	C-index (external validation in 19 cohorts): range 0.685–0.833 for the laboratory-base model, range 0.682-0.813 for the non-laboratory-base model	The model was recalibrated to the 21 WHO regions. The laboratory-based model overpredicted risk in Colombia [99]

*AF* atrial fibrillation, *ASPREE* ASPirin in Reducing Events in the Elderly study, *ASSIGN* Assessing cardiovascular risk using Scottish Intercollegiate Guidelines Network, *AUC* area under the curve, *BMI* body mass index, *CHD* coronary heart disease, *China*-*PAR* Prediction for ASCVD Risk in China, *CONOR* cohort of Norway, *CRP* C-reactive protein, *CVD* cardiovascular disease, *ECG* electrocardiogram, *eGFR* estimated glomerular filtration rate, *EPOCH*-*JAPAN* Evidence for Cardiovascular Prevention from Observational Cohorts in Japan, *HbA1c* hemoglobin A1c, *HDL* high-density lipoprotein, *HF* heart failure, *IHRS* INTERHEART Risk Score, *JALS* Japan Arteriosclerosis Longitudinal Study, *LDL* low-density lipoprotein, *LIFE*-*CVD* LIFEtime-perspective CardioVascular Disease, *LVF* left ventricular hypertrophy, *MI* myocardial infarction, *NORRISK* Norwegian cardiovascular risk prediction model, *PCE* Pooled Cohort Equations, *PREVENT* Predicting Risk of Cardiovascular Disease EVENTs, *RRS* Reynolds Risk Score, *SBP* systolic blood pressure, *SCORE* Systematic COronary Risk Evaluation, *SLE* systemic lupus erythematosus, *TIA* transitory ischemic attack, *uACR* urinary albumin-to-creatinine ratio, *UK* United Kingdom, *US* United States, *WHO* World Health Organization

Most prediction models in Table 3 do not account for competing risks, an important limitation for CVD risk prediction in older adults. Models that account for competing risks include LIFE-CVD2, SCORE2, and SCORE2-Diabetes for Europe [54, 63, 64], NORRISK2 (Norway) [55], and PREVENT (US) [58, 59]. LIFE-CVD2 is a novel lifetime CVD risk prediction tool for adults 35–90 years of age without a history of CVD [54]. The model was calibrated to four CVD risk regions, similar to SCORE2 and SCORE2-OP. In addition to predicting risk, the model allows the estimation of the benefit of lifelong lipid lowering, blood pressure lowering, and smoking cessation. PREVENT is the most updated CVD prediction tool for US adults 30 to 79 years without prior CVD, developed by the American Heart Association [58]. This sex-specific, race-free tool was derived using individual-level data from 25 datasets, comprising more than 3 million people overall. PREVENT allows to estimate 10-year and 30-year risk of CVD, including atherosclerotic CVD and heart failure combined, and atherosclerotic CVD and heart failure, separately.

Most CVD risk prediction tools listed in Table 3 were validated using pooled data from middle-aged and older adults. Therefore, validation results may not be generalizable to older individuals separately. Some models have shown lower discrimination in older adults compared to young individuals, with varying degrees of calibration performance. Below, we describe some specific validation results relevant to older people.

In the external validation of the NORRISK2 (Norway), the area under the receiver operating characteristic curve (UAC) was lower with older age (UAC among men and women 0.62 and 0.64, respectively, for those 65–74 years of age, 0.65 and 0.68 in those 55–64 years of age, and 0.70 and 0.68 in those 45–54 years of age) [55]. Calibration in the older group was considered good based on plots [55]. In the internal validation of the QRISK3 (United Kingdom [UK]), the Harrell’s C statistic among men and women was 0.692 and 0.659, respectively, among participants ≥ 60 years of age, 0.752 and 0.732 for those 40–59 years of age, and 0.747 and 0.781 among those < 40 years of age [60]. The lower discrimination of the QRISK3 in older adults was also present in an external validation using the UK Clinical Practice Research Datalink (Harrell’s C statistic < 0.65 in all subgroups ≥ 65 years of age) [68]. This validation study also reported a substantial overprediction in adults 75–84 years of age [68]. In the SCORE2-Diabetes (Europe), C-indices from the external validation ranged between 0.576 and 0.592 in adults 70–79 years of age and between 0.662 and 0.746 in those 40–49 years of age [64]. Overall, the calibration of the SCORE2-Diabetes was good in older individuals, although it may underpredict risk at the upper end of the age range, particularly among women.

In an analysis using data from adults ≥ 40 years of age in CHS, MESA, Framingham Original, and Framingham Offspring, the discrimination of the PCE (US) was lower in those ≥ 75 years (C-statistic 0.62; 95% CI 0.60, 0.65) versus those < 75 years of age (C-statistic 0.75; 95% CI 0.73, 0.76) [14]. Also, the PCE overestimated risk among adults ≥ 75 years of age [14]. Further information about the performance of CVD risk prediction models for older US adults is provided by two validation studies using data from mostly white individuals ≥ 65 years of age from Australia and the US included in the ASPREE trial [69, 70]. In an ASPREE analysis by Fravel et al., PREVENT, for the prediction of the secondary outcome atherosclerotic CVD, outperformed the PCE [69]. Specifically, the C-statistic in individuals 65–79 and ≥ 80 years of age was 0.793 and 0.854, respectively, for PREVENT, and 0.740 and 0.799, respectively, for the PCE. A higher discrimination for PREVENT versus the PCE was also observed within subgroups by sex, race (white and non-white), and country (Australia and US). The good discrimination of PREVENT and the PCE in the analysis by Fravel et al. contrasts with prior studies showing a decline with older age and needs to be confirmed. In an analysis of the ASPREE trial and the extension observational follow-up, Ganjali et al. compared the performance of the PCE, Framingham general CVD risk score (US), PREDICT1 (New Zealand), and SCORE2-OP (Europe, low-risk region) [70]. The C-statistic was very similar for these four models, including the PCE (range 0.62–0.64 in men and 0.68–0.69 in women). The PCE and the Framingham general CVD risk score overpredicted risk by 20%, while PREDICT1 and SCORE2-OP overpredicted risk by 6%.

### CVD Risk Prediction Models for Secondary Prevention (Recurrent Events)

Few CVD risk prediction models can be used for secondary prevention in older adults (Table 4 [36, 71, 72]). SMART2 was developed for adults 40–80 years of age using data from the Netherlands and calibrated to most world regions, excluding Africa [72]. In an external validation in the UK Biobank, the C-index of SMART2 was 0.663 (95% CI 0.658, 0.668), but results by age were not reported [73]. Although the calibration in the UK Biobank was good, it is unclear which region version of SMART2 was analyzed. PROSPER (northern Europe) was validated using data from SMART [36]. However, the model was recalibrated for external validation, and therefore, the validity of using PROSPER without population-specific recalibration to guide secondary prevention in clinical practice is unclear.

**Table 4:** CVD risk prediction models for secondary prevention (recurrent events)

Model / Year	Country / Region	Target population	Outcome (components)	Horizon	Predictors	Statistical model	Discrimination	Calibration (external)
Framingham subsequent CHD events / 2000 [71]	US	Adults 35–74 years of age with history of CHD or stroke	Recurrent CHD	2-year	Age, total/HDL cholesterol ratio, SBP, diabetes, smoking	Weibull accelerated failure regression. Sex-specific models.	Unavailable	External validation unavailable
PROSPER / 2017 [36]	Northen Europe	Adults 70–82 years of age without chronic kidney disease stages IV or V	Recurrent MACE (MI, stroke or CVD death)	5- and 10-year risk	Sex, age, smoking, diabetes, SBP, LDL and HDL cholesterol, eGFR, number of medications taken, and statin treatment	Accounts for the competing risk of non-CVD death Separate models for older adults with and without a history of CVD.	C-statistic among adults with a history of CVD 0.60 (95% CI 0.56, 0.63) (external validation in the SMART cohort)	The model was recalibrated for external validation in SMART
SMART2 / 2022 [72]	Derived using data from The Netherlands, calibrated to Europe (four regions), Asia excluding Japan, Japan, Australia, North America and Latin America	Adults 40–80 years of age with atherosclerotic CVD	Recurrent atherosclerotic CVD (MI, stroke and CVD death)	10-year	Age, sex, smoking, diabetes, SBP, non-HDL cholesterol, eGFR, CRP, history of atherosclerotic CVD and years since first manifestation	Accounts for the competing risk of non-CVD death	C-statistic (external validation across seven cohorts): 0.605 to 0.772. In an external validation in the UK Biobank, the C-index was 0.663 (95% CI 0.658, 0.668) [73]	There was underestimation of CVD risk in most cohorts before the calibration. Models were recalibrated. Calibration was good in an external validation in the UK Biobank [73]

*AUC* area under the curve, *CHD* coronary heart disease, *CI* confidence interval, *CRP* C-reactive protein, *CVD* cardiovascular disease, *eGFR* estimated glomerular filtration rate, *HDL* high-density lipoprotein, *LDL* low-density lipoprotein, *MACE* major adverse cardiovascular events, *MI* myocardial infarction, *PROSPER* Prospective Study of Pravastatin in the Elderly at Risk, *SBP* systolic blood pressure, *SMART* Secondary Manifestations of ARTerial Disease, *UK* United Kingdom, *US* United States

## Discussion

The present review examines the current state of CVD risk prediction models for older adults. The models identified vary in terms of their specific CVD outcome components, horizon, predictors, and statistical methods. Notably, most models were not developed specifically for older adults, do not consider geriatric conditions as predictors, and do not account for the competing risk from non-CVD-related mortality. While many CVD risk prediction models demonstrated acceptable discriminatory performance for older adults, this was generally lower than in younger individuals. Moreover, evidence on the calibration of these models specifically among older individuals remains limited. Some of the models reviewed are available as online tools (Table 5).

**Table 5:** Online CVD risk prediction tools

Model	Link	Comments
Aus CVD Risk	https://www.cvdcheck.org.au/calculator	
China-PAR	https://cvdrisk.com.cn/ASCVD/Eval	
EPOCH-JAPAN Hisayama Suita Study	https://cran.r-universe.dev/Jcvrisk/doc/manual.html	Available as an R package “jcvrisk”
Framingham general CVD risk score	https://reference.medscape.com/calculator/252/framingham-risk-score-2008#	
Globorisk	https://www.globorisk.org/	
LIFE-CVD2	https://u-prevent.com/calculators/lifeCvd https://hagemanshj.shinyapps.io/LIFECVD2/ (research only)	
NORRISK 2	https://hjerterisiko.helsedirektoratet.no/	
PCE	https://tools.acc.org/ascvd-risk-estimator-plus/#!/calculate/estimate/ http://static.heart.org/riskcalc/app/index.html#!/baseline-risk	
PREDICT	https://www.tewhatuora.govt.nz/for-health-professionals/clinical-guidance/diseases-and-conditions/long-term-conditions/cardiovascular-disease/cvd-risk-assessment-tool	Available as an API
PREVENT	https://professional.heart.org/en/guidelines-and-statements/prevent-calculator	Available also as a package for R (“preventr”) and Stata
QRISK3	https://qrisk.org/three/index.php	
RRS	https://reference.medscape.com/calculator/192/reynolds-cad-risk	
SCORE2 SCORE2-OP	https://www.heartscore.org/en_GB	Available as a package for R “RiskScorescvd”

*API* application programming interface, *China*-*PAR* Prediction for ASCVD Risk in China, *CVD* cardiovascular disease, *EPOCH*-*JAPAN* Evidence for Cardiovascular Prevention from Observational Cohorts in Japan, *PCE* Pooled Cohort Equations, *PREVENT* Predicting Risk of Cardiovascular Disease EVENTs, *RRS* Reynolds Risk Score, *SCORE* Systematic COronary Risk Evaluation, *SCORE*-*OP* SCORE Older Persons

Predicted CVD risk is frequently used to guide treatment initiation or intensification for primary prevention [4–8, 74]. Other potential uses of predicted risk include informing decisions related to treatment discontinuation and guiding secondary prevention therapies, both of which may be more relevant for older versus younger adults [74]. Considering treatment discontinuation could be important for older individuals as this population often has multiple chronic conditions and is prescribed numerous medications (polypharmacy) [75–77], thereby increasing their susceptibility to adverse events, drug-drug interactions, and diminished quality of life [4, 25]. CVD risk prediction is less frequently applied to guide secondary prevention strategies, which could be relevant for older adults, as the prevalence of CVD is high in this population [78]. Given that age ≥ 65 years is a high-risk condition when considering lipid-lowering therapy intensity in people with prevalent CVD [4], most older adults may already be recommended to receive intensive treatment without the need for further risk stratification [79]. Nonetheless, estimating a patient’s CVD risk remains essential for tailoring preventive therapies that are known to reduce cardiovascular morbidity and mortality. The limited number of CVD risk prediction models specifically designed for older adults may be, at least in part, explained by the paucity of evidence-based CVD prevention therapies in this population. For example, Statin Therapy for Reducing Events in the Elderly (STAREE) and PRagmatic EValuation of evENTs And Benefits of Lipid-lowering in oldEr adults (PREVENTABLE) are the first currently ongoing large-scale trials aimed at evaluating the benefits of initiating statin therapy in adults over 70 years of age [80, 81]. Similarly, Statins In The Elderly (SITE) and Statins in Multimorbid Older Adults Without Cardiovascular Disease (STREAM) are the first ongoing trials to assess the clinical impact of statin discontinuation in older individuals [82, 83]. The interest in CVD risk prediction models for older adults is increasing as the evidence from clinical trials assessing therapies in this population is growing [74].

The calculation of a patient’s predicted CVD risk should serve as a starting point for a broader conversation about potential treatment options [4]. Older adults may face competing demands or needs when making clinical decisions [84]. For example, improving quality of life may be a priority over preventing CVD events when the life expectancy is short. Therefore, the incorporation of patient values and preferences, including concerns about adverse events and possible reductions in quality of life, is central to the clinical decision-making process [84]. It is important to remember that these patients’ concerns and values cannot be assessed through CVD risk prediction models. Most CVD risk prediction models designed specifically for older adults, as discussed in this review, provide a 5-year predicted risk. A 5-year predicted risk is often considered more appropriate for older individuals than longer horizons, given their shorter life expectancy [14, 24, 85]. However, most current guidelines use thresholds of 10-year predicted risk to guide treatment decisions [4–7, 86], and converting risk thresholds across different time horizons is not a straightforward linear calculation. Using the PREDICT model in New Zealand, Liang et al. reported that the mean 10-year predicted CVD risk is 2.5 times higher than the mean 5-year predicted risk within the same population [87]. Consequently, thresholds of 10-year predicted risk may need to be about 2.5 times higher than those of 5-year predicted risk in order to identify the same at-risk population [88]. Specifically, a 5-year predicted risk of 5% may correspond to a 10-year predicted risk of approximately 12.5% [88].

Effective communication of predicted CVD risk in a clear and comprehensible manner is an essential component of shared decision-making [89, 90]. However, many patients may struggle to interpret absolute, horizon-specific risk estimates as provided by most CVD risk prediction models [89, 90]. To address this limitation, alternative methods to communicate CVD risk have been developed, including the CVD risk age, defined as “the age of a person with the same estimated risk but with ideal risk factor levels,” [91] and the CVD-free life expectancy, which has been incorporated into the LIFE-CVD2 model [54, 74]. These novel estimates can be particularly useful for young individuals, for whom the estimated 10-year risk may appear numerically low to motivate any action, including lifestyle modifications. Some risk prediction tools also allow the estimation of the expected benefit with the initiation of common preventive interventions like statin therapy or smoking cessation, including the LIFE-CVD2 model (Europe), and the atherosclerotic CVD Risk Estimator Plus, which is based on the PCE (US, available in https://tools.acc.org/ascvd-risk-estimator-plus/#!/calculate/estimate/). The utility of these strategies to communicate CVD risk and inform clinical decision-making in older adults warrants further investigation.

As identified in this review, risk prediction models designed specifically for older adults frequently exhibit lower discrimination performance compared to those for younger individuals. Also, CVD risk prediction models including both younger and older individuals often show a decline in their discrimination performance with older age. Several factors can contribute to explaining this age-related phenomenon, including the high absolute risk of CVD in older populations regardless of the presence of traditional risk factors [78, 92]. Also, older adults with risk factors may represent a very heterogeneous population characterized by the coexistence of individuals who have lived with these risk factors for many decades and others who developed them just at an older age. It has been proposed that incorporating the cumulative exposure to risk factors like high cholesterol or blood pressure as predictors may help to improve the performance of CVD risk prediction models, while avoiding the need for including age, which is non-modifiable [91]. However, obtaining these cumulative estimates may be impractical in most clinical settings. Age is an important risk factor for CVD [78, 92]. For CVD risk prediction models that were developed using mostly data from younger individuals, it would be important to consider non-linear associations between age and CVD risk on the log scale to improve prediction accuracy in the older age groups. An approach that is gaining popularity to avoid making assumptions on the shape of the association between age and CVD risk is using age as the time scale in the regression models (rather than time from baseline), a method that also allows for long-term or lifetime risk estimation [58, 88]. Modeling the effect of older age on other risk factors is also important for improving the performance of CVD risk prediction models that were not developed specifically for older populations [16–20, 91]. This can be achieved by including interaction terms between age and risk factors to account for the attenuation of these associations with older age [16–20, 91].

Improving the discrimination performance of CVD risk prediction models for older adults can be achieved by incorporating more predictors relevant to this population. Many geriatric syndromes and other characteristics more common in older individuals, like low renal function, frailty, depression, or multi-morbidity, are known risk factors for CVD [16, 23, 24]. Factors like the number of medications, polypharmacy, and apathy symptoms (i.e., losing interest in activities, going outside, or trying new things, or feeling without energy) have been shown to improve CVD risk prediction in older adults [16, 35]. Geriatric syndromes are usually ignored by CVD risk prediction models, including those developed specifically for older adults, as presented in this review. Incorporating geriatric syndromes into CVD risk prediction models for older adults may be important not only because of their direct association with a higher CVD risk, but also to account for their effect on competing risks (e.g., elevated non-CVD mortality from conditions like cancer). Lab measurements and markers of subclinical CVD may also be important predictors to be included in risk prediction models for older adults, including N-terminal pro-B-type natriuretic peptide, high-sensitivity cardiac troponin T, coronary artery calcium (CAC) score, ankle-brachial index, carotid intima-media thickness, and valvular calcification, as these may be proximal to the CVD event in their clinical pathway [16, 32, 39, 73, 93]. For example, adding CAC to a refitted Framingham risk prediction model improved the c-statistic from 0.72 to 0.76 in older adults in the Rotterdam study [94]. When developing new CVD risk prediction models, it is important to consider the balance between the improved discrimination performance and the feasibility of measuring more predictors on a large scale. Markers of subclinical CVD, including CAC score, could be used for refining clinical decisions after CVD risk calculation in selected individuals, like those at intermediate risk [95, 96].

**Fig. 1:**
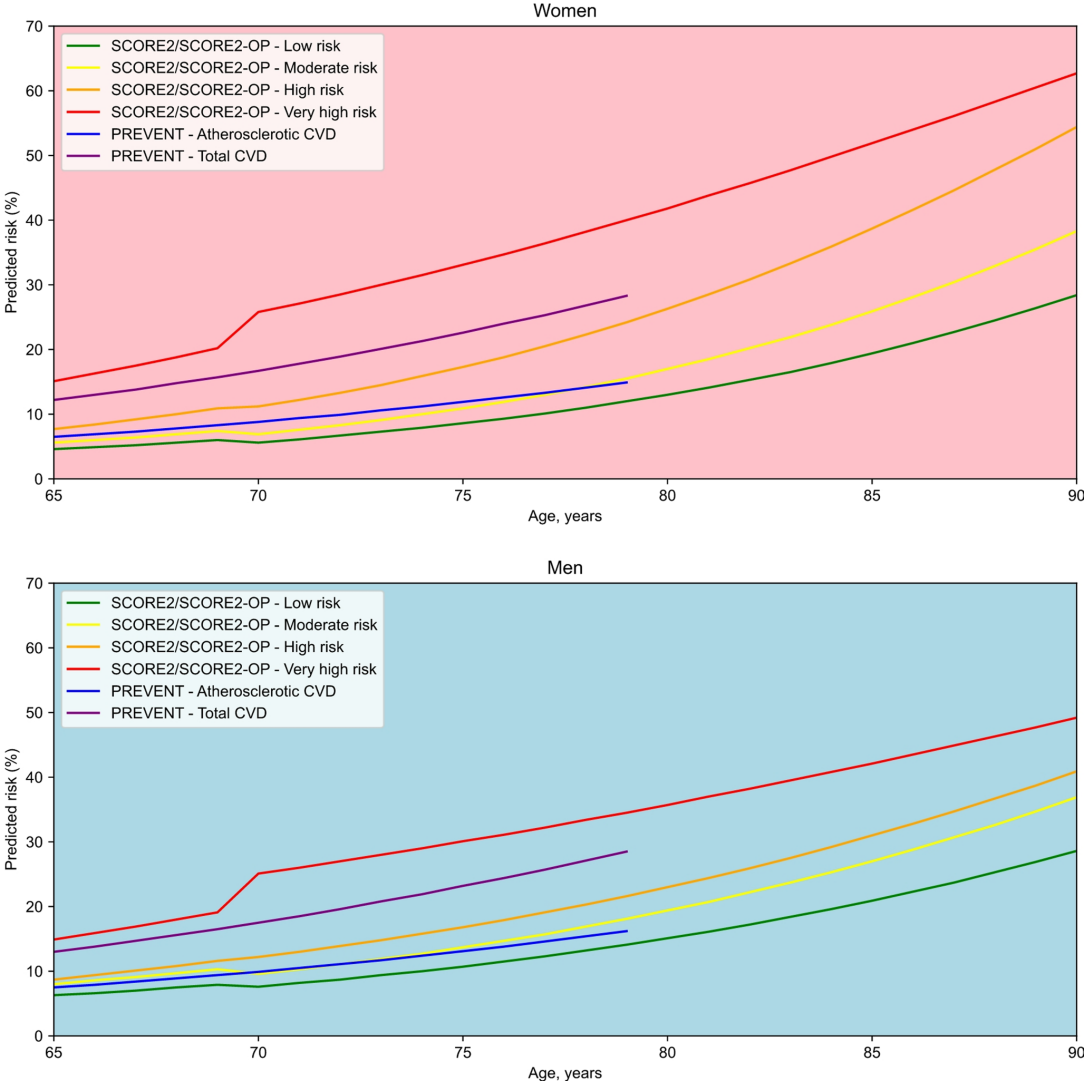
Absolute 10-year risk among women (top panel) and men (bottom panel) 65 to 90 years of age, calculated using selected CVD prediction models. CVD: cardiovascular disease; HDL: high-density lipoprotein; PREVENT: Predicting Risk of Cardiovascular Disease EVENTs; SCORE: Systematic COronary Risk Evaluation; SCORE-OP: Systematic COronary Risk Evaluation, Older Persons; US: United States. The figure shows the absolute 10-year predicted risk for women and men 65 to 90 years of age in four risk regions of Europe (low, moderate, high, and very high risk) using SCORE2 and SCORE2-OP, and in the US using PREVENT. SCORE2 and SCORE2-OP predict the 10-year risk for incident CVD, defined as myocardial infarction, stroke, or CVD death. Risk estimates from SCORE2 and SCORE2-OP were calculated in R using the “RiskScorescvd” package and assuming a systolic blood pressure of 130 mm Hg, total cholesterol of 170 mg/dL (4.4 mmol/L), and HDL cholesterol of 50 mg/dL (1.3 mmol/L), in a non-smoking individual without diabetes. The primary outcome of PREVENT is the 10-year risk of incident total CVD, defined as coronary heart disease, stroke, or heart failure. For comparison, we also calculated the 10-year predicted risk for the secondary outcome of atherosclerotic CVD, defined as coronary heart disease or stroke. Risk estimates from PREVENT were calculated in R using the “preventr” package and assuming a systolic blood pressure of 130 mm Hg, total cholesterol of 170 mg/dL (4.4 mmol/L), HDL cholesterol of 50 mg/dL (1.3 mmol/L), estimated glomerular filtration rate of 70 ml/min/1.73 m^2^, and body mass index of 25 Kg/m^2^, in a non-smoking individual without diabetes and not receiving antihypertensive or statin therapy

The calibration performance of CVD risk prediction models relates directly to their use to inform clinical decisions based on specific risk thresholds [4, 8]. In this context, it is important to acknowledge that CVD risk prediction models that ignore the competing risk of non-CVD death may overestimate CVD risk, and this overestimation will be larger for older versus younger adults [16, 22, 97]. As discussed in the current review, CVD risk prediction models have been developed for high- and low-risk regions, and therefore, it is important to apply CVD risk prediction models that are appropriately calibrated for the target population. The Fig. 1 presents the estimated 10-year CVD risk for women and men aged 65 to 90 years, as calculated using SCORE2 and SCORE2-OP across the four European risk regions and using PREVENT for both total CVD and atherosclerotic CVD among US adults. As shown, the predicted risk may vary substantially across regions and the type of CVD outcome predicted. Notably, the predicted 10-year risk of atherosclerotic CVD using PREVENT overlaps with those estimates for the moderate-risk region in Europe. The current review emphasizes findings related to the calibration performance of CVD risk prediction models overall. However, the calibration is more relevant around the thresholds of predicted risk used to guide clinical decision-making. Many CVD risk prediction models show poor calibration at the higher end of the risk distribution, on which inaccuracies are unlikely to change clinical decisions [98]. Patients and clinicians need to keep in mind that a predicted risk is not an exact measurement. Therefore, thresholds should not be considered as rigid cut-offs but rather as part of a continuum. Clinical judgment incorporating additional information, such as the presence of risk factors or markers of subclinical CVD not included in the risk prediction models, is essential when making a treatment decision [4].

It has been suggested that the development of new CVD risk prediction models may not be necessary to account for changes in risk factors and CVD incidence over time [9, 26]. Instead, existing models could be periodically updated through recalibration [9, 26]. This strategy can be applied with SCORE2, SCORE2-OP, and LIFE-CVD2, which were specifically designed to allow their recalibration using updated data on regional risk factor distributions and CVD incidence rates [37, 54, 63]. Recalibration, however, cannot improve discrimination. Therefore, the development of new CVD risk prediction models for older adults may be justified by the need to improve the discrimination performance in this population.

## Conclusions

This review identifies CVD risk prediction models specifically designed for older adults, as well as those applicable to this population, highlighting some opportunities to enhance the accuracy and clinical utility of CVD risk assessment in later life. Despite certain limitations, existing models offer valuable tools to support evidence-based decision-making in the care of older individuals.

## Key References

- Si F, Liu Q, Yu J. A prediction study on the occurrence risk of heart disease in older hypertensive patients based on machine learning. BMC Geriatr. 2025;25(1):27. doi: 10.1186/s12877-025-05679-1.
  - Innovative risk prediction model for Chinese adults ≥60 years of age without heart disease with hypertension developed using machine learning techniques.
- Belahnech Y, Ródenas-Alesina E, Muñoz M, Verdu-Rotellar JM, Sao-Avilés A, Urio-Garmendia G, et al. Systematic Coronary Risk Evaluation 2 for Older Persons: 10 years risk validation, clinical utility, and potential improvement. Eur J Prev Cardiol. 2025;32(7):527-36. doi: 10.1093/eurjpc/zwae383.
  - This study validated the SCORE2-OP model in a representative Mediterranean cohort aged ≥65 years, showing good discrimination, better in women, and good calibration, better in men, for 10-year cardiovascular risk. Incorporating valvular calcification into the model significantly improved predictive performance and may help reduce unnecessary treatments in older adults.
- Hageman SHJ, Kaptoge S, de Vries TI, Lu W, Kist JM, van Os HJA, et al. Prediction of individual lifetime cardiovascular risk and potential treatment benefit: development and recalibration of the LIFE-CVD2 model to four European risk regions. Eur J Prev Cardiol. 2024;31(14):1690-9. doi: 10.1093/eurjpc/zwae174.
  - This study presents the LIFE-CVD2 model, a calibrated tool for estimating lifetime cardiovascular risk and treatment benefit across four European risk regions, showing good discrimination.
- Khan SS, Matsushita K, Sang Y, Ballew SH, Grams ME, Surapaneni A, et al. Development and Validation of the American Heart Association's PREVENT Equations. Circulation. 2024;149(6):430-49. doi: 10.1161/CIRCULATIONAHA.123.067626.
  - Development and validation of the most updated CVD risk prediction model for US adults 30 to 79 years of age by the American Heart Association. It includes several innovative features compared to prior CVD risk prediction models for US adults: includes the CVD outcome component of heart failure, incorporates kidney and metabolic predictors, is race-free, and accounts for the competing risk of non-CVD death.
- Fravel MA, Ernst ME, Woods RL, Orchard SG, Ganjali S, Wetmore JB, et al. Performance of the American Heart Association PREVENT Cardiovascular Risk Equations in Older Adults. Circ Cardiovasc Qual Outcomes. 2025:e011719. doi: 10.1161/circoutcomes.124.011719.
  - This study evaluated the AHA PREVENT risk equations in adults aged ≥65 years and found that PREVENT outperformed the PCE in predicting 10-year atherosclerotic CVD risk, including in those aged ≥80 years.
- Ganjali S, Lotfaliany M, Tonkin A, Nelson MR, Reid CM, McNeil JJ, et al. Predictive performance of cardiovascular disease risk prediction models in older adults: a validation and updating study. Heart. 2025. doi: 10.1136/heartjnl-2025-325665.
  - This study found that updating commonly used CVD risk models originally developed for middle-aged adults significantly improved their predictive accuracy and clinical utility in healthy adults aged 70 years or older.
- Hageman SHJ, McKay AJ, Ueda P, Gunn LH, Jernberg T, Hagström E, et al. Estimation of recurrent atherosclerotic cardiovascular event risk in patients with established cardiovascular disease: the updated SMART2 algorithm. Eur Heart J. 2022;43(18):1715-27. doi: 10.1093/eurheartj/ehac056.
  - This study presents the development and validation of the SMART2 risk score, an updated tool for estimating the 10-year risk of recurrent atherosclerotic CVD events accounting for competing risks. This risk prediction model was calibrated for most regions in the world, excluding Africa.

## Data Availability

No datasets were generated or analysed during the current study.
